# New species and new records of bryozoans from Galicia (NW Spain)

**DOI:** 10.1080/00222933.2019.1582815

**Published:** 2019-03-27

**Authors:** Oscar Reverter-Gil, Javier Souto, Juan E. Trigo

**Affiliations:** a Museo de Historia Natural da Universidade de Santiago de Compostela, Santiago de Compostela, Spain; b Institut für Paläontologie, Fakultät für Geowissenschaften, Geographie und Astronomie, Geozentrum, Universität Wien, Wien, Austria; c Departamento de Zooloxía e Antropoloxía Física, Facultade de Bioloxía, Universidade de Santiago de Compostela, Santiago de Compostela, Spain; d Grupo de Estudo do Medio Mariño (GEMM), A Coruña, Spain

**Keywords:** Iberian Peninsula, NE Atlantic, *Antarctothoa*, *Dentiporella*, *Fenestrulina*, *Schizotheca*

## Abstract

Although the bryozoological fauna of Galicia (NW Spain) is probably the best known of the whole Iberian Peninsula, and perhaps one of the better known in Europe, new studies continue to provide new knowledge. A new species, *Schizotheca galaica* sp. nov., is described. Eleven species are newly recorded in Galicia: *Aetea longicollis, Parellisina curvirostris, Copidozoum planum, Glabrilaria corbula, Haplopoma sciaphilum, Schizomavella* (*Schizomavella*) *mamillata, Fenestrulina asturiasensis, Fenestrulina barrosoi, Buffonellaria muriella, Schizotheca divisa* and *Dentiporella saldanhai*; two of them (*B. muriella* and *S. divisa*) are also reported for the first time in Iberian waters; four others (*Antarctothoa galaica, F. asturiasensis, F. barrosoi* and *D. saldanhai*) are reported for the first time since their original descriptions, and SEM images of *A. longicollis* and *F. asturiasensis* are provided for the first time. Moreover, the range of geographical distribution of some species is expanded: the record of *S. divisa* is the southernmost to date, while the records of *S. mamillata, F. barrosoi* and *D. saldanhai* are the most northerly to date. The presence of other four species in Galician waters is confirmed and we document the permanence and range extension of two species recently introduced into our waters (*Tricellaria inopinata* and *A. galaica*).

urn:lsid:zoobank.org:pub:F8D0ABEF-026E-4FC8-A947-6484249519FA

## Introduction

Bryozoans are key components of the benthos, presenting a wide diversity and encrusting all types of hard, permanent or ephemeral substrates. Studies on the bryozoological fauna of Galicia began with the publication of the results of the campaign by the *Travailleur* carried out by Jullien (1882, 1883), a very late date compared with the long bryozoological tradition in other areas such as the British Isles. Moreover, the first records of intertidal bryozoans did not appear until well into the twentieth century, when Barroso (1923) published the results of a study of material collected in Marín (Ría de Pontevedra). The second contribution in this field had to wait for another 50 years, when Carrada (1973) published data from the Ría de Vigo. From the 1980s on, however, a continuous series of works increased our knowledge on the Galician bryozoological fauna. The publication, 20 years later, of an annotated check-list (Reverter-Gil and Fernández-Pulpeiro 2001) that compiled data of more than 260 species, crowned this effort. This revealed Galicia to be the most diverse area of the Iberian waters as far as bryozoans are concerned, and one of the best-known areas in Europe. It is also worth mentioning the importance of the group in the Galician benthos, since the high diversity of bryozoans in some rías (e.g. the Ría de Ferrol with 143 known species or the Ría de Vigo with 122 species) defines these areas as having the greatest number of species per area of the entire Atlantic.

During the past years we have been gathering unpublished data from various sites along our coasts. This has yielded new data for nearly 100 species. In the present work we collate the most interesting data on 19 of them, including: the description of a new species of the genus *Schizotheca* Hincks, 1877; the first records of 11 species for Galician waters, two of which [*Buffonellaria muriella* Berning and Kukliński, 2008 and *Schizotheca divisa* (Norman, 1864)] are also reported for the first time in Iberian waters; four others [*Antarctothoa galaica* (César-Aldariz, Fernández-Pulpeiro and Reverter-Gil, 1999), *Fenestrulina asturiasensis* Álvarez, 1992, *Fenestrulina barrosoi* Álvarez, 1993 and *Dentiporella saldanhai* Souto, Reverter-Gil and Fernández-Pulpeiro, 2010] are reported for the first time since their original descriptions. SEM photos of *Aetea longicollis* Jullien in Jullien and Calvet 1903, and *F. asturiasensis* are here included for the first time. The presence of other four species in Galician waters is confirmed, previously doubtful due to different reasons; and we confirm the permanence and range extension of species recently introduced into our waters, such as *Tricellaria inopinata* d’Hondt and Occhipinti Ambrogi, 1985 and *A. galaica*.

In addition, the record of *S. divisa* becomes the southernmost of the species to date, whereas the finding of *Schizomavella* (*Schizomavella*) *mamillata* (Hincks, 1880) in our waters confirms the presence of the species in Atlantic waters and it becomes the most northerly to date.

## Materials and methods

**Figure 1.**
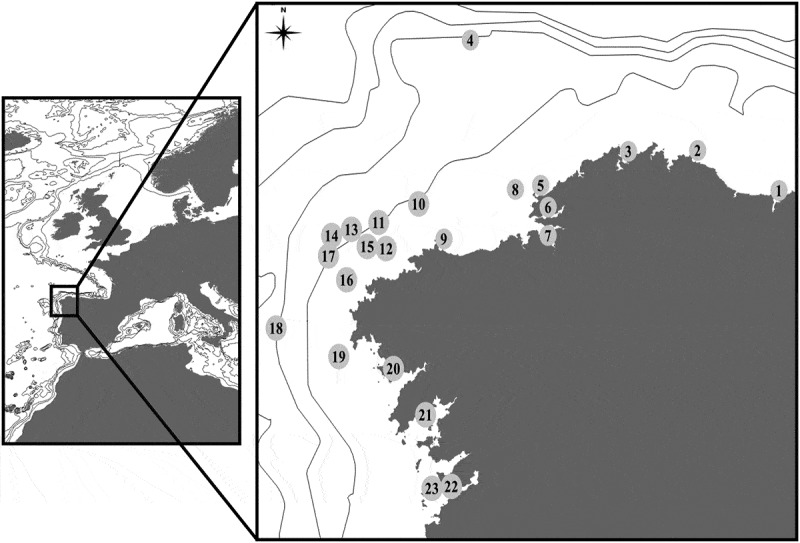
Sampling localities in Galician waters. See also Table 1.

The samples studied here were collected all along the Galician coast (NW Spain), in 43 localities, from the intertidal to 594 m depth (Figure 1; Table 1).

**Table 1.** Sampling localities in Galicia (see also Figure 1). (EFP: E. Fernández-Pulpeiro; JC: J. Cremades; JET: Juan E. Trigo; JLR: J.L. Redondo; JS: J. Souto; MNHN: Muséum National d’Histoire Naturelle, Paris; ORG: O. Reverter-Gil).

No	Locality	N	W	Depth	Date	Coll./ID
1a	Ría de Ribadeo: R25	43.53694°	07.03611°	0	7 August 1979	EFP/ORG
1b	Ría de Ribadeo: R26	43.54000°	07.03750°	0	8 August 1979	EFP/ORG
1c	Ría de Ribadeo: R28	43.55000°	07.03583°	0	28 February 1979	EFP/ORG
1d	Ría de Ribadeo: R30	43.55222°	07.03694°	0	30 October 1977	EFP/ORG
2	Xove	43.71300°	07.46840°	0	21 February 2008	JC/EFP
3	Ría de Ortigueira	43.70889°	07.83639°	0	April 2017	ORG
4	*Thalassa* Y428	44.19667°	08.67667°	500–522	4 September 1972	MNHN/ORG
5a	Prior: El Porto	43.56000°	08.30300°	10	4 May 2003	JS
5b	Prior: A Cova	43.56006°	08.31672°	6–14	18 June 2008	JS
5c	Prior: A Cova	43.56006°	08.31672°	15	16 August 2010	JS
6a	Ría de Ferrol: Redonda	43.46389°	08.26333°	8	June 2004	ORG
6b	Ría de Ferrol: Cetárea Vella	43.45592°	08.29939°	4	12 March 2004	JS/ORG
6c	Ría de Ferrol: A Graña	43.47817°	08.25845°	0	27 October 2010	JS
6d	Ría de Ferrol: Sta. Lucía	43.46139°	08.25000°	0	2 April 2013	EFP/ORG
7	Ría de Ares: Carnoedo	43.38083°	08.26361°	8	21 June 2010	JS
8		43.54167°	08.43722°	94	10 January 2003	JET/ORG
9	Malpica	43.32444°	08.81722°	0	4 November 1983	EFP/ORG
10		43.47806°	08.97083°	172	23 November 2002	JET/ORG
11		43.39667°	09.18139°	186	2 November 2003	JET/ORG
12		43.28194°	09.14333°	95	14 January 2003	JET/ORG
13		43.36639°	09.32778°	223	29 January 2002	JET/ORG
14		43.33917°	09.39556°	249	14 January 2003	JET/ORG
15		43.29139°	09.20667°	143	6 November 2004	JET/ORG
16		43.14500°	09.35361°	133	14 January 2003	JET/ORG
17		43.25000°	09.45000°	220	27 September 2002	JET/ORG
18		42.93333°	09.72833°	594	-	JLR/ORG
19		42.80833°	09.39500°	128	May 1997	JLR/ORG
20a	Ría de Muros: Louro	42.75583°	09.10444°	0	July 2005	ORG
20b	Ría de Muros: P. San Francisco	42.75778°	09.07472°	0	July 2005	ORG
20c	Ría de Muros: Baroña	42.69194°	09.02833°	0	17 March 1984	EFP/ORG
21a	Ría de Arousa: N of O Grove	42.49167°	08.90833°	6	November 1978	EFP/ORG
21b	Ría de Arousa: O Grove	42.49610°	08.85920°	0	7 March 2011	JS
21c	Ría de Arousa: Tragobe	42.51850°	08.82740°	0	7 March 2011	JS
21d	Ría de Arousa: Cambados	42.52106°	08.84173°	3	30 July 2008	JS
21e	Ría de Arousa: Illa de Arousa	42.55306°	08.93361°	40	19 August 2003	JET/ORG
22a	Ría de Vigo: Cangas	42.25417°	08.83333°	5	15 October 2010	JS/ORG
22b	Ría de Vigo: Canido	42.19361°	08.79917°	0	20 January 1984	EFP/ORG
22c	Ría de Vigo: V34	42.23889°	08.79639°	16	16 September 1986	EFP/ORG
23a	Cíes Islands: Illa de Monteagudo	42.24251°	08.90188°	11	22 August 2012	JS/ORG
23b	Cíes Islands: Illa de Monteagudo	42.23871°	08.89947°	6	22 August 2012	JS/ORG
23c	Cíes Islands: P. das Margaridas	42.23532°	08.89828°	5	22 August 2012	JS/ORG
23d	Cíes Islands: P. Figueiras	42.23028°	08.89944°	0	August 2008	ORG
23e	Cíes Islands: Illa de Monteagudo	42.22883°	08.89511°	6	22 August 2012	JS/ORG
23f	Cíes Islands: Illa San Martiño	42.20450°	08.90697°	5	23 August 2012	JS/ORG

In the intertidal, samples from the localities 1, 3, 6d, 9, 20, 22b and 23d were collected on natural substrates, whereas specimens from locality 2 were collected on an experimental panel, and material from localities 6c, 21b and 21c were collected on artificial structures in marinas. The material was initially conserved in alcohol or dry.

Shallow-water specimens were collected by SCUBA (localities 5, 6b, 7, 21d, 22a and 23a–c, e, f) or by dredge (localities 6a, 21a and 22c) during systematic sampling campaigns in different rías. As in the case of the intertidal specimens, they were initially fixed in alcohol or preserved dry.

Another group of samples, from localities 8, 10–19 and 21e, were collected during fishing activities mainly with bottom trawls and fishtraps from near-shore fishing boats. During the use of these fishing gears rocks, shells, and corals among other substrates with bryozoans were collected. Most of these samples were dried for conservation. Finally, one sample collected at st. Y428 of the campaign *Thalassa* (here named st. 4) and currently stored in the Muséum National d’Histoire Naturelle, Paris (MNHN), was studied.

Data on the substrates, when known, are presented in the material examined section of each species.

Samples were sorted and examined in the lab using stereomicroscopes. Selected specimens were dried for study by scanning electron microscopy (SEM). FEI Inspect S50 SEM and Zeiss EVO LS15, from the University of Vienna and from the University of Santiago de Compostela, respectively, were used to take photographs of uncoated material with a back-scattered electron detector in low-vacuum mode. Optical photos were taken with a Nikon D90 camera. Measurements were taken with the software ImageJ® on SEM photographs.

For comparative purposes, some type material held in the MNHN and the Museo Nacional de Ciencias Naturales, Madrid (MNCN) has been revised. The material newly collected during the present work has been stored in the bryozoan collection at the Museo de Historia Natural of the USC (MHNUSC-Bry).

## Results

Order **CHEILOSTOMATIDA** Busk, 1852

**Figure 2.**
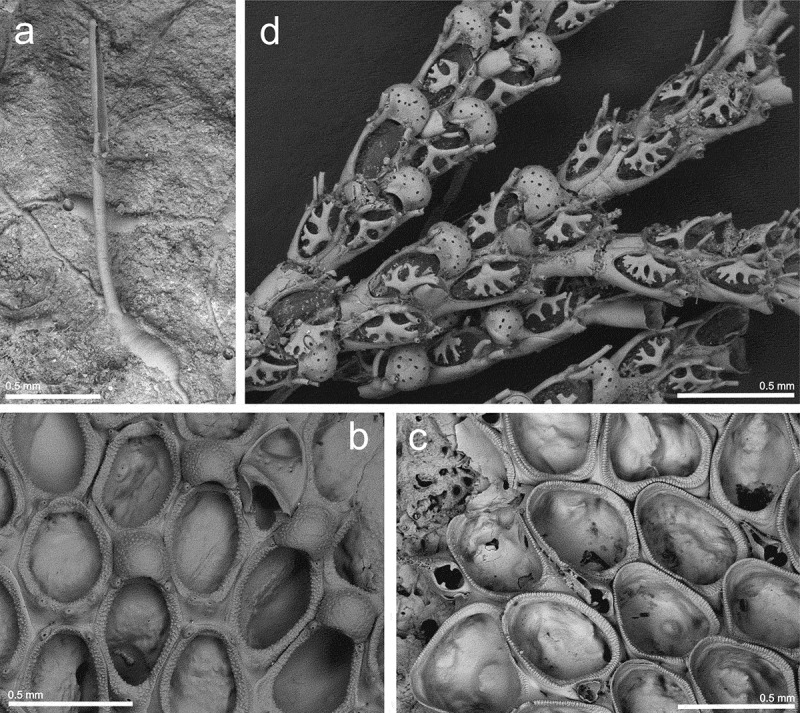
(a) A zooid of *Aetea longicollis* (st. 19; MHNUSC-Bry 420); (b) ovicelled zooids and avicularium of *Parellisina curvirostris* (st. 14; MHNUSC-Bry 500); (c) autozooids and avicularia of *Copidozoum planum* (st. 13; MHNUSC 10091); (d) portion of a colony of *Cradoscrupocellaria ellisi* (st. 1a; MHNUSC-Bry 90f).

Suborder **INOVICELLINA** Jullien, 1888Family **AETEIDAE** Smitt, 1868Genus ***Aetea*** Lamouroux, 1812***Aetea longicollis*** Jullien in Jullien and Calvet, 1903(Figure 2(a))

*Aetea longicollis* Jullien in Jullien and Calvet, 1903: 32, pl. 2, fig. 7.

### 

#### Material examined

St. 19: several colonies on a whale bone (42.80833°N, 09.39500°W, 128 m depth; MHNUSC-Bry 418, 420, 509) (Figure 2(a)).

#### Description

Colony encrusting, uniserial, ramifying, white when dead, colour alive unknown. Autozooids with a basal dilatation and a stem arising from its distal part. Length of the dilatation, which is covered by transverse lines, about one third of stem length; width about 0.12–0.18 (mean 0.15) mm. Stems long and slender, about 2 mm long by 0.07 mm wide. Distal portion not widened, bearing the narrow frontal membrane, 0.70 mm long by 0.06 mm wide, with a terminal operculum. Stem smooth, lightly punctate, distal portion around the membranous area highly punctate.

#### Remarks

*Aetea longicollis* is a rarely reported species whose distribution is not well known due to frequent confusions with other species of the genus, although it seems to be present both in the NE Atlantic and the Mediterranean. The species was originally described by Jullien (in Jullien and Calvet 1903) from material collected off Luarca (Asturias, N Spain) at 134 m depth. It had also been reported in Catalonia (Zabala 1986) at 5–20 m depth, on vertical walls, in small caves and on detritic bottoms, especially on algae and the seagrass *Posidonia*, although this record seems doubtful because this represents a completely different habitat.

*Aetea longicollis* had not been previously reported in Galicia, although the species *Aetea lineata*, described by Jullien (1882) from a single zooid devoid of peristome, collected near Galician coasts, could actually correspond to *A. longicollis* (see Reverter-Gil and Fernández-Pulpeiro 2001).

Suborder **FLUSTRINA** Smitt, 1868Family **CALLOPORIDAE** Norman, 1903Genus ***Parellisina*** Osburn, 1940***Parellisina curvirostris*** (Hincks, 1862)(Figure 2(b))

*Membranipora curvirostris* Hincks 1862: 29; Hincks, 1880: 153, pl. 20, figs. 5, 6.*Parellisina curvirostris*: Hayward and Ryland 1998: 182, fig. 52.

#### Material examined

St. 14: several ovicelled colonies on eroded fragments of *Reteporella* sp. (43.33917°N, 09.39556°W, 249 m depth; MHNUSC-Bry 500) (Figure 2(b)).

#### Description

Colony encrusting, unilaminar. Autozooids oval or irregular, 0.511–0.716 (mean 0.574) mm long by 0.330–0.410 (mean 0.363) mm wide, separated by shallow grooves. Basal wall thin, lightly calcified. Lateral wall raised. Cryptocyst narrow, granular. Gymnocyst smooth, poorly developed, more evident in the proximal end of the autozooid. Short spines, roughly conical, around the opesia: two distal, even in ovicellate zooids, and one to three proximal. Small basal pore chambers present. Large vicarious avicularia, 0.123–0.470 (mean 0.327) mm long by 0.265–0.288 (mean 0.277) mm wide, with a triangular rostrum raised, directed distally and curved to one side; rostrum with an oval area. Proximal end with a rounded opesia, surrounded by a poorly developed granular cryptocyst. Both areas separated by a pair of condyles. Each avicularium with a small distal kenozooid, roughly triangular. Ovicell hyperstomial, small, wider than long, 0.116–0.220 (mean 0.180) mm long by 0.190–0.221 (mean 0.211) mm wide. Ectooecium membranous; entooecium granular, with some small peripheral pseudopores. Orifice narrow, not closed by the operculum.

#### Remarks

According to Hayward and Ryland (1998), *P. curvirostris* has a wide distribution in warm temperate and subtropical waters. In Iberian waters it does not seem to be a very frequent species: along the Mediterranean coast it has been reported only in Catalonia and the Balearic Islands (Gautier 1962; Zabala 1993), while in the Atlantic coast only in the Ibero-Moroccan Bay at 150 m depth (Harmelin and d’Hondt 1992). Besides, the species *Membranipora guernei*, described by Jullien and Calvet (1903) from 135 m depth off the Cape Peñas (Asturias), is probably a junior synonym of *P. curvirostris* according to Prenant and Bobin (1966). The present record is therefore the first one of the species in Galician waters.

Genus ***Copidozoum*** Harmer, 1926***Copidozoum planum*** (Hincks, 1880)(Figure 2(c))

*Membranipora plana* Hincks, 1880: 81, pl. 11, fig. 2.*Copidozoum planum*: Hayward and Ryland 1998: 180, figs. 47C, 51A.

#### Material examined

St. 13: a small colony on a dead fragment of *Porella compressa* (43.36639°N, 09.32778°W, 223 m depth; MHNUSC 10091, together with more species, including the holotype of *Schizotheca galaica* sp. nov. – see below) (Figure 2(c)).

#### Description

Colony encrusting, unilaminar, with oval autozooids, large, 0.405–0.518 (mean 0.478) mm long by 0.321–0.381 (mean 0.353) mm wide, lightly calcified. Gymnocyst reduced to a small proximal area. Cryptocyst narrow, finely granular. Spines absent. Interzooidal avicularia 0.233–0.296 (mean 0.268) mm long, with a straight, narrow rostrum parallel-sided, directed distally, and a semicircular opesia proximal to a pair of condyles. Ovicell not observed.

#### Remarks

*Copidozoum planum* seems to be a warm-temperate species with a circum-subtropical distribution. In Europe it is frequent in the Mediterranean and extends north to near the English Channel. In Iberian waters it has been reported all along the Mediterranean coast, but in the Atlantic coast it was recorded only in the Ibero-Moroccan Bay at 580 m depth by Harmelin and d’Hondt (1992) and in the Algarve (Souto et al. 2014). Therefore, the present record of *C. planum* is the first one for the Galician coast.

Family **CANDIDAE** d’Orbigny, 1851Genus ***Cradoscrupocellaria*** Vieira, Spencer Jones and Winston, 2013***Cradoscrupocellaria ellisi*** (Vieira and Spencer Jones, 2012)(Figure 2(d))

*Scrupocellaria ellisi* Vieira and Spencer Jones, 2012: 34, figs. 4, 18–23, 25, 27 (cum syn.).

*Cradoscrupocellaria ellisi*: Vieira et al. 2013: 41, fig. 20.

#### Material examined

St. 1a: several colonies (Ría de Ribadeo: R25, Pta. Cabanela, 43.53694°N, 07.03611°W, intertidal; MHNUSC-Bry 90e, f) (Figure 2(d)).

St. 1b: several colonies (Ría de Ribadeo: R26, Muelle de Porcillán, 43.54000°N, 07.03750°W, intertidal; MHNUSC-Bry 90b).

St. 1c: several colonies (Ría de Ribadeo: R28, M. del cargadero a Castillo de S. Damián, 43.55000°N, 07.03583°W, intertidal; MHNUSC-Bry 90d).

St. 1d: several colonies (Ría de Ribadeo: R30, P. Guitón, 43.55222°N, 07.03694°W, intertidal).

St. 6b: several colonies (Ría de Ferrol: Cetárea Vella, 43.45592°N, 08.29939°W, 4 m depth; MHNUSC-Bry 595).

St. 9: several ovicelled colonies (Malpica, 43.32444°N, 08.81722°W, intertidal).

St. 20a: many colonies on red seaweeds (Ría de Muros: Louro, 42.75583°N, 09.10444°W, intertidal).

St. 20c: several colonies (Baroña, 42.69194°N, 09.02833°W, intertidal; MHNUSC-Bry 90c).

St. 21a: several colonies (Ría de Arousa: O Grove, 42.49167°N, 08.90833°W, 6 m depth).

St. 21e: (Porto de Xufre, Illa de Arousa, 42.55306°N, 08.93361°W, 40 m depth, MHNUSC-Bry 506).

St. 22a: several colonies on *Cliona celata* (Ría de Vigo: Cangas, Pta. Creixiña, Nerga, 42.25417°N, 08.83333°W, 5 m depth; MHNUSC-Bry 361).

St. 22b: several colonies (Ría de Vigo: Canido, 42.19361°N, 08.79917°W, intertidal).

St. 22c: several colonies (Ría de Vigo, V-34, 42.23889°N, 08.79639°W, 16 m depth).

St. 23a: several colonies (Cíes Islands: Furna de Monteagudo, 42.24251°N, 08.90188°W, 11 m depth).

St. 23b: several ovicelled colonies, and juveniles, on algae (Cíes Islands: Illa de Monteagudo, 42.23871°N, 08.89947°W, 6 m depth; MHNUSC-Bry 622, 624).

St. 23c: several colonies (Cíes Islands: Praia das Margaridas, 42.23532°N, 08.89828°W, 5 m depth; MHNUSC-Bry 626).

St. 23d: several colonies with embryos (Cíes Islands: Playa de Figueiras, 42.23028°N, 08.89944°W, intertidal; MHNUSC-Bry 605).

St. 23e: several young colonies on shells and algae (Cíes Islands: Punta Muxieiro, 42.22883°N, 08.89511°W, 6 m depth; MHNUSC-Bry 598, 604, 610, 613, 631).

St. 23f: several ovicelled colonies (Cíes Islands: Illa de San Martiño, 42.20450°N, 08.90697°W, 5 m depth; MHNUSC-Bry 611).

#### Remarks

*Scrupocellaria ellisi* was recently described by Vieira and Spencer Jones (2012) for several specimens previously identified as *Scrupocellaria reptans* (Linnaeus, 1758). Both species are distinguished by very few characters not always easy to see: the presence in *S. ellisi* of smooth rhizoids and stouter scuta with 8–13 stout projections at distal tips, against 6–9 in *S. reptans*, and the size of oecial pseudopores, smaller in *S. ellisi* than in *S. reptans*. The next year, both species were transferred to the new genus *Cradoscrupocellaria* Vieira et al., 2013. Geographical distribution of *C. reptans* was limited to the British Isles, since most of its previous records were assigned to *C. ellisi*, a species widespread in the north-east Atlantic (North Sea, British Channel, Irish Sea, Celtic Sea), the Adriatic, Tasmania (probably introduced), and perhaps present also in the western Mediterranean (see Vieira et al. 2013).

*Cradoscrupocellaria reptans* was until now considered to be a frequent and abundant species, reported all along the Galician coast, from Ribadeo to Vigo, collected from the intertidal down to 25 m depth, growing mostly on algae and shells (Reverter-Gil and Fernández-Pulpeiro 2001, as *Scrupocellaria reptans*). We have revised collection material previously identified as *S. reptans*, as well as other material newly collected, all coming from different localities of Galicia, and we believe that it must be identified as *C. ellisi*. Nonetheless, as we have not been able to verify all the previous records of *S. reptans* in Galicia individually, we cannot rule out that this species, or other similar ones, were also present in our waters.

Genus ***Tricellaria*** Fleming, 1828***Tricellaria inopinata*** d’Hondt and Occhipinti Ambrogi, 1985

*Tricellaria inopinata* d’Hondt and Occhipinti Ambrogi, 1985: 35–46, fig. 2, 3.

#### Material examined

St. 2: many colonies on an experimental panel (Xove: 43.71300°N, 07.46840°W, intertidal).

St. 3: many colonies on algae (Ría de Ortigueira: Playa de Morouzos, 43.70889°N, 07.83639°W, intertidal; MHNUSC-Bry 253, 254).

St. 5b: on *Sargassum* (Prior: A Cova, 43.56006°N, 08.31672°W, 6–14 m depth).

St. 6c: (Ría de Ferrol: A Graña, 43.47817°N, 08.25845°W, intertidal; MHNUSC-Bry 280, 308).

St. 6d: (Ría de Ferrol: Ensenada de Santa Lucía, 43.46139°N, 08.25000°W, intertidal).

St. 7: Ría de Ares: Carnoedo, 43.38083°N, 08.26361°W, 8 m depth.

St. 20b: many colonies on *Sargassum* (Ría de Muros: beach of San Francisco, 42.75778°N, 09.07472°W, intertidal).

St. 21b: (Ría de Arousa: O Grove, 42.49610°N, 08.85920°W, intertidal; MHNUSC-Bry 313).

St. 21c: (Ría de Arousa: Pantalanes del muelle de Tragove, 42.51850°N, 08.82740°W, intertidal).

St. 21d: (Ría de Arousa: Cambados, 42.52106°N, 08.84173°W, 3 m depth).

St. 23b: several colonies on seaweeds (Cíes Islands: Illa de Monteagudo, 42.23871°N, 08.89947°W, 6 m depth; MHNUSC-Bry 625).

#### Remarks

*Tricellaria inopinata* was collected for the first time in Iberian waters in samplings carried out in 1996 in the Ría de Ribadeo. This finding most likely represents a recent introduction (*c*. 1990) from the Venetian lagoon together with clams for cultivation (Fernández-Pulpeiro et al. 2002). The next time that the species was collected in Galicia was at several localities in the Ría de Vigo in 1998 (Soto García et al. 2002), although at that time it was not possible to verify whether its presence was the result of an independent introduction or the expansion of the species from the north coast of Galicia. In fact, *T. inopinata* was collected again in the Ría de Ribadeo in 1999 and in 2000, confirming its acclimatisation to the area, and also in 2000 in the Ría de Muros. The new records included here confirm the colonisation of the entire Galician coast by this species. *Tricellaria inopinata* may even appear in very high abundances in some places, as is the case for the Island of San Vicente (Ría de Ortigueira) (st. 3), or the beach of San Francisco (Ría de Muros) (st. 20b).

**Figure 3.**
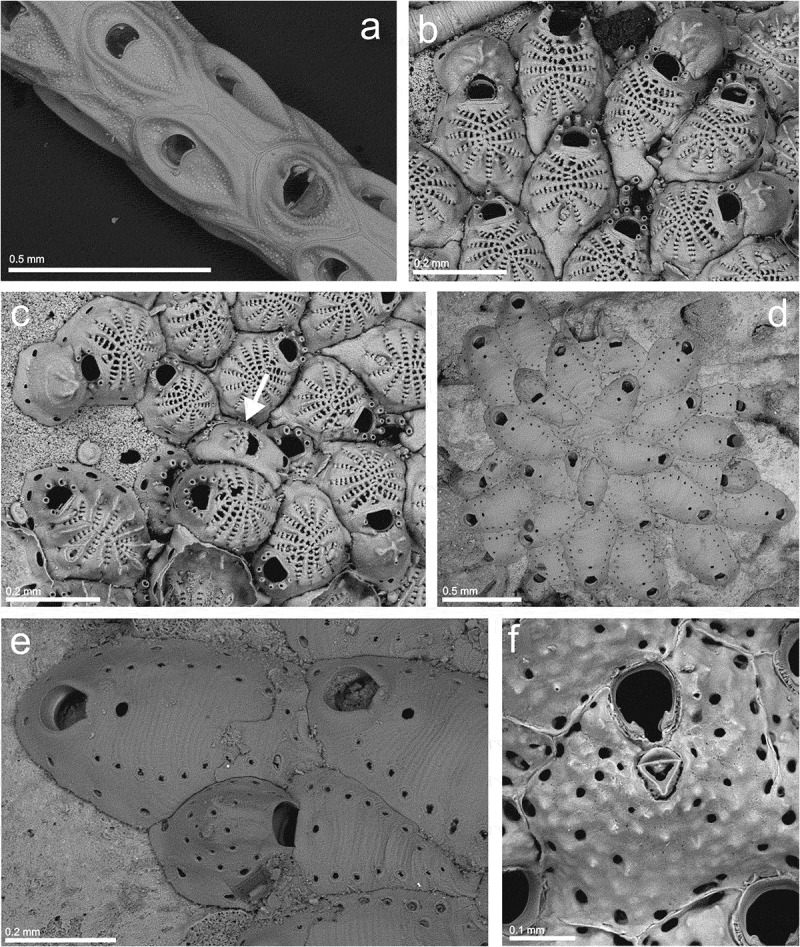
(a) Autozooids and avicularium of *Cellaria salicornioides* (st. 16; MHNUSC-Bry 515); (b) ovicelled and non-ovicelled zooids of *Glabrilaria corbula*; top right, a partially covered zooid of *Cribrilaria bifida* (d’Hondt, 1970) (st. 6a; MHNUSC-Bry 658); (c) same, a kenozooid formed by regeneration of a broken zooid (arrow) (perhaps the ancestrula?); bottom left, a zooid of *C. bifida*; (d) a colony of *Haplopoma sciaphilum* (st. 19; MHNUSC-Bry 520); (e) same, autozooids and ovicelled zooid; (f) an autozooid of *Schizomavella* (*Schizomavella*) *mamillata* (st. 12; MHNUSC-Bry 657).

Family **CELLARIIDAE** Fleming, 1828Genus ***Cellaria*** Ellis and Solander, 1786***Cellaria salicornioides*** Lamouroux, 1816(Figure 3(a))

*Cellaria salicornioides* Lamouroux 1816: 127; Hayward and Ryland 1998: 308, figs. 104A, 105D, 108.

#### Material examined

St. 16: fragments of colonies (43.14500°N, 09.35361°W, 133 m depth; MHNUSC-Bry 515) (Figure 3(a)).

#### Remarks

*Cellaria salicornioides* is a widespread species in the Mediterranean, ranging northwards to British coasts and southwards to the Canaries. Off the Iberian Peninsula it had been reported in many localities both along Mediterranean and Atlantic coasts. In waters near Galicia, however, *C. salicornioides* had only been reported more than 130 years ago, by Jullien (1882, as *Salicornaria johnsoni* Busk) at 1037 m depth. The original material of this record was not found in the MNHN collections in Paris, so it has not been checked. The present record confirms the presence of *C. salicornioides* on the Galician coast.

Family **CRIBRILINIDAE** Hincks, 1879 Genus ***Glabrilaria*** Bishop and Househam, 1987***Glabrilaria corbula*** (Bishop and Househam, 1987) (Figure 3(b,c))

*Puellina* (*Glabrilaria*) *corbula* Bishop and Househam, 1987: 52, figs. 82–91; Harmelin and Arístegui 1988: 530, figs. 26–29.

*Puellina corbula*: Hayward and Ryland 1998: 326, figs. 114A, B.

*Glabrilaria corbula*: Rosso et al. 2018: 407.

#### Material examined

St. 6a: one ovicellate colony on a broken shell (Ría de Ferrol: Redonda, 43.46389°N, 08.26333°W, 8 m depth; MHNUSC-Bry 658) (Figure 3(b,c)).

#### Description

Colony encrusting, unilaminar, small, consisting of only 19 autozooids, round or sub-oval, 0.191–0.291 (mean 0.238) mm long by 0.155–0.194 (mean 0.175) mm wide. Lateral and proximal gymnocyst narrow to broad, but with proximal or proximolateral extensions between neighbouring zooids. Pericyst lightly calcified, without central umbo, with 10–12 costae; each costa with a broad, ascending portion at periphery of pericyst leading to a narrower portion, nearly horizontal, at the flat central region of shield, both portions separated by a low tubercle bearing a very small pelmatidium. Six to eight intercostal reniform pores, generally smaller in proximal costae. Orifice of non-ovicellate zooid D-shaped, 0.035–0.047 (mean 0.039) mm long by 0.044–0.055 (mean 0.050) mm wide, proximal edge straight. Orifice of ovicellate zooid width 1.2 times that of orifice of non-ovicellate zooid. Seven distal oral spines, four in ovicellate zooids, without inwardly directed apophyses. Apertural bar with pelmatidium each side of midline, without tubercles. One or two median sub-oral lacunae between proximal margin of orifice and first row of intercostal pores. Ovicell of category C (see Bishop and Househam 1987), 0.092–0.127 (mean 0.113) mm long by 0.136–0.166 (mean 0.151) mm wide; length 0.7 times that of frontal shield; without a median suture but with up to four ridges in a more or less radiating pattern. Kenozooid observed once, with an incomplete cribrimorph frontal shield of costae in radiating pattern. Formed by regeneration of a broken zooid (perhaps the ancestrula?) (Figure 3(c)). Avicularium not present in our material.

#### Remarks

*Glabrilaria corbula* was described by Bishop and Househam (1987) from material collected in the English Channel and NE Ireland at 73–106 m depth. The following year, Harmelin and Arístegui (1988) reported this species from deep waters in the Ibero-Moroccan Bay at 518–524 m depth and the French Mediterranean coast at 130–300 m depth, and also in cryptic habitats of the NW Mediterranean (submarine caves and small cavities in biogenic rocks) at 6–30 m depth. *Glabrilaria corbula* was reported again by Harmelin and d’Hondt (1992) from the Ibero-Moroccan Bay at 135–521 m depth and from the Alboran Sea at 145–170 m depth, and then again by De Blauwe (2009) from the southern North Sea.

Harmelin and Arístegui (1988) pointed out that the Mediterranean material differs from the British one mainly by the frontal shield, which is less prominent and without marked peripheral protuberances because the bases of the costae are low with a small tubercle bearing a pelmatidium, instead of being steeply raised with a tall and rounded tubercle or short crest. Furthermore, deep-water material from the Mediterranean and Ibero-Moroccan Bay differs from the Mediterranean littoral specimens only by a tendency to lack avicularia. For these reasons, Harmelin and Arístegui (1988) considered that specimens from the Ibero-Moroccan Bay, despite being geographically Atlantic, belong to the Mediterranean population of the species. We only have a single, small colony of *G. corbula*, collected at 8 m depth in the Ría de Ferrol (NE Atlantic). Nonetheless, despite the scarcity of the material collected, the shape of the costae as well as of the whole pericyst indicates that it is more closely related to the Mediterranean population of the species than to the British one. The absence of avicularia in our material could relate it to the deep-water material, but we believe that this lack may simply reflect the scarcity of material studied. This scarcity (only a juvenile colony) may also explain the smaller size of our material compared with records by Harmelin and Arístegui (1988) or Hayward and Ryland (1998).

The Ría de Ferrol presents a very high bryodiversity in spite of its small size (up to 143 species according to Reverter-Gil and Fernández-Pulpeiro 2001 and unpublished data). The present record of *G. corbula* increases the number of species of *Puellina s.l*. reported in the ría to nine (see Reverter and Fernández 1996). Furthermore, the ría houses the only Iberian records of *Puellina nana* Reverter-Gil and Fernández-Pulpeiro, 2007a, *Puellina modica* Bishop and Househam, 1987, *Puellina directa* Bishop and Househam, 1987 and *G. corbula*. For three of these species, those records are the shallowest ones to date: 20 m for *P. modica*, and 8 m for both *G. corbula* and *P. directa*, which were collected here at the same station (MHNUSC-Bry 647).

Family **HIPPOTHOIDAE** Busk, 1859 Genus ***Antarctothoa*** Moyano, 1987***Antarctothoa galaica*** (César-Aldariz, Fernández-Pulpeiro and Reverter-Gil, 1999)

*Celleporella galaica* César-Aldariz et al., 1999: 53, figs. 2, 3.

*Antarctothoa galaica*: Hughes et al. 2008: 373.

#### Material examined

St. 5a: several colonies with embryos growing on algae (Prior: El Porto, 43.56000°N, 08.30300°W, 10 m depth; MHNUSC-Bry 360).

#### Remarks

To date, *A. galaica* was known only from the localities along the coast of Lugo reported in its original description (César-Aldariz et al. 1999) because the species was not reported again. Therefore, the present record is the first one since the original description of the species and confirms that *A. galaica*, although probably introduced in NW Spain from the Southern Hemisphere via shipping (Hughes et al. 2008), is still present here more than 20 years later.

Family **HAPLOPOMIDAE** Gordon in De Blauwe, 2009Genus ***Haplopoma*** Levinsen, 1909***Haplopoma sciaphilum*** Silén and Harmelin, 1976(Figure 3(d,e))

*Haplopoma sciaphilum* Silén and Harmelin, 1976: 61; Hayward and Ryland 1999: 310, fig. 143.

#### Material examined

St. 19: two small ovicelled colonies on a whale bone (42.80833°N, 09.39500°W, 128 m depth; MHNUSC-Bry 520) (Figure 3(d,e)).

#### Description

Colony encrusting, forming a small patch. Zooids dimorphic. Autozooids ovoid, elongated, 0.494–0.574 (mean 0.536) mm long by 0.230–0.352 (mean 0.288) mm wide, separated by narrow grooves. Frontal wall vitreous, translucent, convex, with thin transverse grooves, without umbo. Ascopore circular, about 0.20 mm diameter, joined to the zooidal orifice by a suture visible by transparency. A single series of marginal pores, large and circular. Communication via small pore chambers. Orifice linguiform, 0.066–0.095 (mean 0.80) mm long by 0.072–0.091 (mean 0.081) mm wide; rounded distally, with a lunula; proximal border straight, narrow, with acute-angled corners. Ovicellate zooids shorter, 0.226–0.366 (mean 0.288) mm long by 0.181–0.328 (mean 0.238) mm wide, with smaller ascopore. Orifice D-shaped, 0.058 mm long by 0.082 mm wide. Ovicell acleithral, subglobular, 0.130–0.202 (mean 0.169) mm long by 0.187–0.226 (mean 0.203) mm wide, with irregularly scattered pseudopores and peripheral pore chambers. Ancestrula 0.20 mm long by 0.12 mm wide, partially overgrown in examined material, with orifice keyhole-shaped, both parts asymmetric, separated by a pair of small pointed condyles. Anter with a lunula. Initial budding distinctive: unilateral, asymmetric, with the first daughter autozooid directed backwards and partially covering the ancestrula.

#### Remarks

According to Hayward and Ryland (1999), *H. sciaphilum* is relatively common in dark environments and deep waters. It has been reported from west Sweden, through Britain to the Mediterranean and Adriatic seas. In the Iberian Peninsula, however, it has been previously recorded only twice: in submarine caves in Sagres (S Portugal) by Boury-Esnault et al. (2001) and in Gipuzkoa (N Spain) at 3–10 m depth by d’Hondt (1988). However, this last record seemed doubtful because *H. sciaphilum* is characterised by the lack of an umbo, whereas d’Hondt (1988) referred to a well-developed umbo in his material. The revision of the reference material deposited in the MNHN (MNHN-IB-2008–15376 and 15383) confirms that this record actually corresponds to ovicelled colonies of *Haplopoma impressum* (Audouin, 1826) growing on algae. The present record is then the second one for the Iberian waters and the first one for the Galician coast.

The size of the autozooids of the present material is similar to the British one (Hayward and Ryland 1999); the latter authors stated that autozooids of *H. sciaphilum* are smaller in the Mediterranean.

Family **BITECTIPORIDAE** MacGillivray, 1895Genus ***Schizomavella*** Canu and Bassler, 1917***Schizomavella* (*Schizomavella*) *mamillata*** (Hincks, 1880)(Figure 3(f))

*Schizoporella linearis* var. *mamillata* Hincks, 1880: 248.

*Schizomavella mamillata*: Hayward and McKinney 2002: 59, fig. 27 A–C.

#### Material examined

St. 12: a colony on a brachipod shell (43.28194°N, 09.14333°W, 95 m depth; MHNUSC-Bry 657) (Figure 3(f)).

#### Description

Colony encrusting, multilaminar, developing as a small crust. Autozooids rectangular or irregularly polygonal, 0.601–0.854 (mean 0.722) mm long by 0.274–0.355 (mean 0.315) mm wide, in radial series in growing edge, randomly orientated in areas with frontal budding, separated by fine, raised sutures; frontal shield convex, nodular, irregularly perforated by few pores, plus a row of conspicuous areolar pores. Primary orifice drop-shaped, longer than wide, 0.113–0.132 (mean 0.123) mm long by 0.095–0.105 (mean 0.100) mm wide, with a small, U-shaped sinus occupying one-third of the proximal border. Condyles distinctive, thick, longer than wide, with free distal edge sharply cusped, and reaching the edges of the medial notch, defining a deep, V-shaped sinus at binocular. Two or three oral spines, vestigial, covered by secondary calcification. Avicularium diagnostic: median suboral, monomorphic, with triangular rostrum, 0.099–0.109 (mean 0.103) mm long by 0.069–0.078 (mean 0.073) mm wide, directed proximally and slightly acute to frontal plane; crossbar complete, with strong median columella, palate with trifoliate foramen. Ovicell not present in the single colony studied.

#### Remarks

The present material differs in only two characters from other records of *S. mamillata* (see e.g. Hayward and McKinney 2002; Reverter-Gil et al. 2015): the species typically presents a peristome developed as a thin rim, most pronounced on each side of sinus. In our material the peristome is absent, but it seems to be broken, perhaps eroded (Figure 3(f)). On the other hand, the avicularium of our material is much smaller than the avicularium in Mediterranean material, but nonetheless similar to that observed in colonies from south Portugal (see Souto et al. 2010 fig. 16B), so this is perhaps a character defining the Atlantic material of the species.

*Schizomavella mamillata* seems to be common throughout the shallow waters of the Mediterranean (Hayward and McKinney 2002), but it is also present in two Atlantic localities along the Portuguese coast: Foz do Douro, intertidal, and in the Algarve at 20 m depth (Reverter-Gil et al. 2014). The present record is thus the first one of *S. mamillata* for the Galician coast, and is also the northernmost of the species to date.

**Figure 4.**
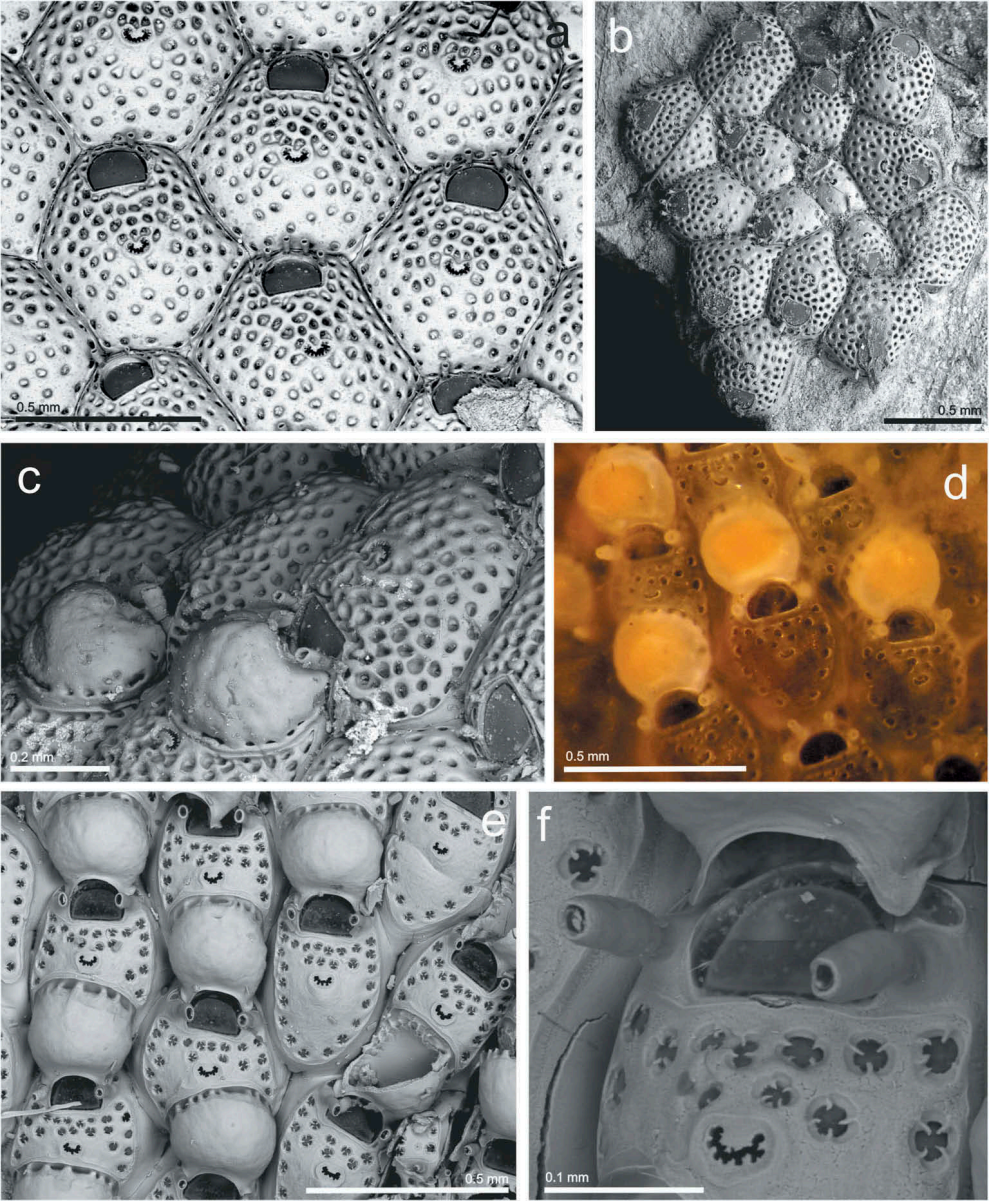
(a) Holotype of *Fenestrulina asturiasensis* (MNCN 25.03/187); (b) a young colony of *F. asturiasensis* (st. 19; MHNUSC-Bry 404); (c) same, ovicelled zooids (st. 12; MHNUSC-Bry 517); (d) ovicelled zooids of *Fenestrulina barrosoi* with embryos (st. 5c; MHNUSC-Bry 639); (e) same, ovicelled zooids; (f) same, detail showing the pseudopores, the ascopore and the oral spines.

Family **MICROPORELLIDAE** Hincks, 1879Genus ***Fenestrulina*** Jullien, 1888***Fenestrulina asturiasensis*** Álvarez, 1992(Figure 4(a–c))

*Fenestrulina asturiasensis* Álvarez, 1992: 727, fig. 1, 2.

#### Material examined

St. 12: a ovicellate colony on *Reteporella* sp. (43.28194°N, 09.14333°W, 95 m depth; MHNUSC-Bry 517) (Figure 4(c)).

St. 17: several colonies on coral (43.25000°N, 09.45000°W, 220 m depth; MHNUSC-Bry 516).

St. 19: several colonies on a whale bone (42.80833°N, 09.39500°W, 128 m depth; MHNUSC-Bry 404, 518) (Figure 4(b)).

#### Comparative material examined

MNCN 25.03/187: Holotype of *Fenestrulina asturiasensis*, Cape Peñas (Asturias, N Spain) 120 m depth, 03/06/1991 (Figure 4(a)).

#### Description

Colony encrusting, forming small unilaminar patches. Autozooids roughly hexagonal, 0.430–0.588 (mean 0.511) mm long by 0.358–0.540 (mean 0.444) mm wide, in alternating series, separated by distinct grooves. Frontal wall smooth, convex, evenly perforated by circular, non-stellate pores, each one located at the bottom of a small, short cavity. In young zooids pores are lacking in the area proximal to ascopore, except marginal ones. Ascopore in the centre of the zooid; lumen crescentic, with small delicate denticulations. Large basal pore-chambers present. Primary orifice D-shaped, wider than long, 0.096–0.126 (mean 0.110) mm long by 0.137–0.152 (mean 0.143) mm wide. Four to five short cylindrical oral spines, reduced to two in ovicellate zooids. Ooecium acleithral, subglobular, prominent, wider than long, recumbent on distal succeeding zooid, with smooth surface and a single peripheral series of basal pores.

#### Remarks

*Fenestrulina asturiasensis* was described by Álvarez (1992) for two colonies collected at 120 m depth off Cape Peñas (Asturias, northern Spain). As far as we are aware, the species has not been reported again since then. Therefore, this is not only the first record of *F. asturiasensis* for the Galician coast, but also the first one since the original description of the species. We also provide the first SEM images of the species, both of the type material and the newly collected material. The holotype of *F. asturiasensis* was growing on a brachiopod shell, but the new specimens collected here were found on *Reteporella* sp., corals and on a whale bone.

***Fenestrulina barrosoi*** Álvarez, 1993(Figure 4(d–f))

*Fenestrulina barrosoi* Álvarez, 1993: 831, fig. 1.

#### Material examined

St. 5c: several small colonies on algae (Prior: A Cova, 43.56006°N, 08.31672°W, 15 m depth; MHNUSC-Bry 639) (Figure 4(d–f)).

#### Comparative material examined

MNCN-25.03/280: Holotype of *Fenestrulina barrosoi*, Isla de Alborán, 45–52 m.

#### Description

Colony encrusting, forming a flat unilaminar crust on algae. Autozooids oval, 0.357–0.501 (mean 0.435) mm long by 0.284–0.344 (mean 0.313) mm wide, in alternating series separated by distinct grooves. Frontal shield convex, smooth, lightly calcified. A single series of marginal pseudo-stellate pseudopores, with 2–4 (often 3 or 4) radii, not joined in the centre; two series of pseudopores passing between the primary orifice and the ascopore. Two distolateral depressions separated by the two mid-distal spines, each one with 1–2 pseudopores. Large basal pore-chambers present. Primary orifice D-shaped, 0.078–0.102 (mean 0.091) mm long by 0.122–0.148 (mean 0.135) mm wide. Four to five oral spines, the proximal pair particularly thickened, club-shaped, present in ovicellate zooids. Ascopore in the distal half of autozooid, with smooth rim oval to reniform separated from primary orifice by 0.07–0.10 mm; lumen crescentic, with small delicate denticulations. Ooecium acleithral, globular, prominent, 0.198–0.230 (mean 212) mm long by 0.209–0.261 (mean 242) mm wide, with smooth surface surrounded by a single series of marginal pores. Ancestrula tatiform, with 10 peripheral spines. Daughter zooids budded mid-distally and distolaterally, small. Embryos, yellow in life, present in August.

#### Remarks

*Fenestrulina barrosoi* was described by Álvarez (1993) from material collected at 28–52 m depth close to the Alboran Island (SW Mediterranean). As for *F. asturiasensis* (see above) the present record is the first one for the Galician coast, but also the first one since its original description. The present record also proves for the first time the presence of *F. barrosoi* in the Atlantic Ocean.

Álvarez (1993) indicated that a difference between *F. barrosoi* and *Fenestrulina malusii* (Audouin, 1826) was that the former grows on basal structures of sea-grasses in the Alboran Sea whereas the later was collected encrusting shells. Nevertheless, in Galicia *F. barrosoi* was found growing on algae, a substrate also used by *F. malusii* in the Atlantic Ocean (Hayward and Ryland 1999; Reverter-Gil and Fernández-Pulpeiro 2001). Therefore, the substrate does not seem to be a good diagnostic character.

**Figure 5.**
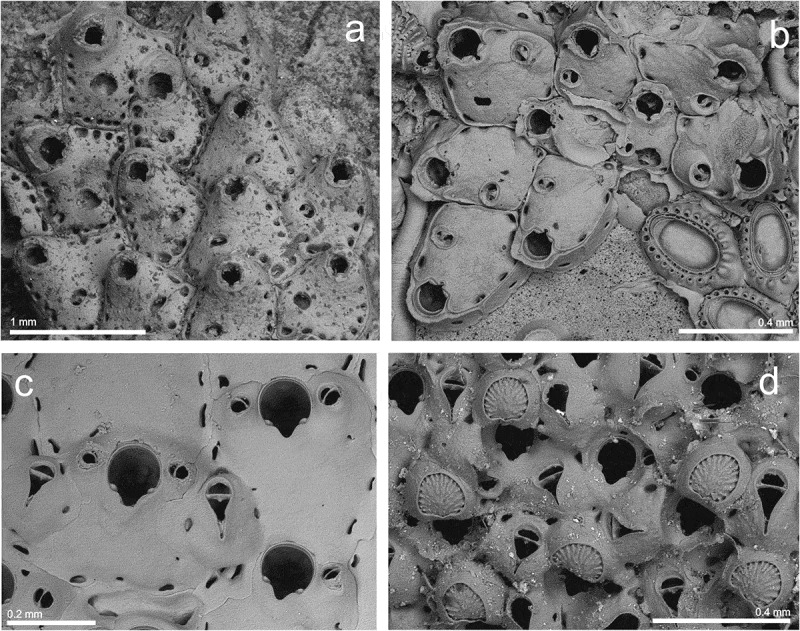
(a) Ovicelled and non-ovicelled zooids of *Herentia thalassae* (st. 18; MHNUSC-Bry 183); (b) young colony of *Herentia hyndmanni* (st. 6a; MHNUSC-Bry 658); (c) young autozooids of *Buffonellaria muriella* with adventitious and vicarious avicularia (st. 10; MHNUSC-Bry 492); (d) same, ovicelled zooids and vicarious avicularia (st. 11; MHNUSC-Bry 491).

Family **ESCHARINIDAE** Tilbrook, 2006Genus ***Herentia*** Gray, 1848***Herentia thalassae*** David and Pouyet, 1978 (Figure 5(a)

*Herentia* (*Herentia*) *thalassae thalassae* David and Pouyet 1978, part: 172, pl. 1, fig. 1.

*Herentia thalassae*: Berning et al. 2008: 1524, fig. 4.

#### Material examined

St. 4: one colony on a stone (44.19667°N, 08.67667°W: *Thalassa* Y428, 500–522 m depth; MNHN IB-2013–578).

St. 18: several eroded, ovicelled colonies on a mineralised whale bone (42.93333°N, 09.72833°W, 594 m depth (Reverter-Gil and Fernández-Pulpeiro 2001 as *Escharina hyndmanni*: MHNUSC-Bry 183, 184) (Figure 5(a)).

#### Comparative material examined

MNHN 9982: Holotype of *Herentia* (*Herentia*) *thalassae thalassae, Thalassa* U851, 520–530 m.

#### Remarks

*Herentia thalassae* has been recently redescribed by Berning et al. (2008), who establish the characters to differentiate it from the very similar species *Herentia hyndmanni* (Johnston, 1847) and indicate the difficulty to confirm the previous identifications without examining the original material. *Herentia thalassae* was described from the station *Thalassa* U851, coming from north of A Coruña at 520–530 m depth [MNHN 9982: Holotype; David and Pouyet 1978 as *Herentia* (*Herentia*) *thalassae thalassae*]. This reference was overlooked by Reverter-Gil and Fernández-Pulpeiro (2001).

*Herentia hyndmanni* was reported from Galicia by d’Hondt (1974), but of all the material reported only the sample MNHN 7619 (station *Thalassa* U825, from west of Vigo at 480–520 m depth), seems to have been preserved. This sample was included and figured by Berning et al. (2008) in their redescription of *H. thalassae*. Furthermore, *H. thalassae* was originally described in other of the stations reported by d’Hondt (1974) (*Thalassa* U851). And finally, the unpublished locality where this species is reported in the present work (*Thalassa* Y428) is in the same area as those referred to by d’Hondt (1974). Therefore, it is possible that all the records by d’Hondt (1974) from northern Galicia identified as *H. hyndmanni* actually correspond to *H. thalassae*.

Finally, the material reported by Reverter-Gil and Fernández-Pulpeiro (2001) as *Escharina hyndmanni* off Fisterra at 128 m depth could not be revised, but the material collected at 594 m depth is revised here and its identification is corrected to *H. thalassae*.

***Herentia hyndmanni*** (Johnston, 1847)(Figure 5(b))

*Lepralia hyndmanni* Johnston, 1847: 306, pl. 54, fig. 6.

*Escharina hyndmanni*: Hayward and Ryland 1999: 232 (part), figs. 95C, D, 98A, B, *non* C.

*Herentia hyndmanni*: Berning et al. 2008: 1516, fig. 1.

#### Material examined

St. 6a: a young colony on a broken shell (Ría de Ferrol: Redonda, 43.46389°N, 08.26333°W, 8 m depth; MHNUSC-Bry 658) (Figure 5(b)).

#### Remarks

According to Reverter-Gil and Fernández-Pulpeiro (2001, as *Escharina hyndmanni*), this species would have been cited in Galicia in three areas: in the Ría de Ferrol at 15–20 m depth; in several stations of the *Thalassa* north of A Coruña, between 380 m and 530 m depth; and in the Fisterra area, at 128 m and 594 m depth. However, following the redescription of the genus *Herentia* by Berning et al. (2008) there were doubts about whether *H. hyndmanni* is really present in Galicia or not.

As already indicated in the previous species, the records from the *Thalassa* campaign and off Fisterra possibly correspond entirely to *H. thalassae*. However, Berning et al. (2008, p. 1526) indicate that both species can coexist, so the presence of *H. hyndmanni* in these waters cannot be completely ruled out.

The material originally reported from the Ría de Ferrol as *E. hyndmanni* (see Reverter Gil 1995) has not been conserved, so it could not be revised. Note on the one hand, however, that the 7 colonies collected at 15–20 m depth, some of them ovicellated, were formed by essentially flat autozooids and ovicells, which contrasts with the distally elevated autozooids in *H. thalassae* provided with ooecias rising well above colony surface (see Figure 5(a,b)). On the other hand, a juvenile colony collected in Ferrol in 2004 in a station close to the original ones, at 8 m depth (Figure 5(b)), corresponds well to the description of *H. hyndmanni* by Berning et al. (2008). We can therefore confirm that this species exists in Galician waters. The presence of this species in Galicia is not surprising, considering that according to Berning et al. (2008) *H. hyndmanni* occurs off the western coasts of the British Isles and off southern Portugal, and Galicia is halfway between both areas.

Family **CELLEPORIDAE** Johnston, 1838Genus ***Buffonellaria*** Canu and Bassler, 1927***Buffonellaria muriella*** Berning and Kukliński, 2008(Figure 5(c,d))

*Schizoporella biaperta* (Michelin, 1848): Hincks 1880: 255, pl. 40, fig. 7–9.

*Buffonellaria divergens* (Smitt, 1873): Hayward and Ryland 1999: 356, fig. 167; fig. 183C, D (as *Stephanollona armata*).

*Buffonellaria muriella* Berning and Kukliński, 2008: 549, figs. 1A, 2A, 8A–F.

#### Material examined

St. 10: two small colonies on a broken urchin test and a gastropod shell (43.47806°N, 08.97083°W, 172 m depth; MHNUSC-Bry 492, 493) (Figure 5(c)).

St. 11: one ovicelled colony (43.39667°N, 09.18139°W, 186 m depth; MHNUSC-Bry 491) (Figure 5(d)).

#### Description

Colony encrusting, unilaminar, multiserial. Autozooids elongate oval to roughly hexagonal, 0.374–0.441 (mean 0.404) mm long by 0.309–0.378 (mean 0.340) mm wide, separated by shallow grooves obscured by secondary calcification; frontal wall slightly convex, smooth, initially with few pseudopores, becoming imperforate during later ontogeny; four to seven slit-like, marginal, areolar pores. Primary orifice variable in shape, 0.127–0.150 (mean 0.136) mm long by 0.104–0.129 (mean 0.118) mm wide; anter roughly oval in outline; poster with a deep and rounded V-shaped sinus, occupying about two thirds of the total width; condyles conspicuous, relatively broad, blunt, shorter than the proximal margins. Small oral avicularia single or paired, rarely absent, 0.047–0.070 (mean 0.059) mm long by 0.035–0.053 (mean 0.042) mm wide, situated on a slightly raised cystid, frontal plane at an acute angle to colony surface; rostrum semi-elliptical, directing proximally or proximolaterally; crossbar complete, thin. Additional larger avicularia present in older areas of the colony, 0.116–0.190 (mean 0.147) mm long by 0.053–0.093 (mean 0.073) mm wide, arising from marginal pores of the autozooid, situated on large cystid; rostrum elongate triangular, narrowing distal to crossbar, becoming slender and parallel-sided distally, pointing in various directions; crossbar complete, occasionally with a small median columella. Ooecium globular, prominent, 0.164–0.203 (mean 0.182) mm long by 0.182–0.207 (mean 0.192) mm wide, slightly broader than long. Ectooecium a broad smooth band, exposed entooecium roughly semicircular, relatively flat, marked with 14–18 (frequently 17) radiating tubercular ribs slightly thickening towards margin.

#### Remarks

*Buffonellaria muriella* was recently introduced by Berning and Kukliński (2008) for several previous European records made as *Schizoporella biaperta* (Michelin, 1848) and as *Buffonellaria divergens* (Smitt, 1873). Confirmed records of *B. muriella* extend from the Irish Sea to the English Channel and the Netherlands (Berning and Kukliński 2008; De Blauwe 2009), and also in the southern part of the Adriatic and perhaps Naples (Berning and Kukliński 2008). In the Iberian Peninsula there are several records of *S. biaperta* and *B. divergens*, from both the Atlantic and Mediterranean coasts, but without complete descriptions and SEM figures it is not possible to deduce to which species those records belong. Thus, the present record of *B. muriella* confirms the presence of this species in Iberian waters.

Our material (only three colonies) differs from the original description mainly by the biometries: autozooids, avicularia (both oral and frontal) and ovicells are clearly smaller in the Galician material, while primary orifices have a similar size, though also slightly smaller. Size of avicularia greatly differs from Adriatic material of the species, which presents even larger oral and frontal avicularia. However, the Galician material is similar to the Adriatic material in its smaller condyles, more ovicell ribs (frequently 17) and a less conspicuous columella in frontal avicularia. Our material could represent a combination of Atlantic and Mediterranean characters.

**Figure 6.**
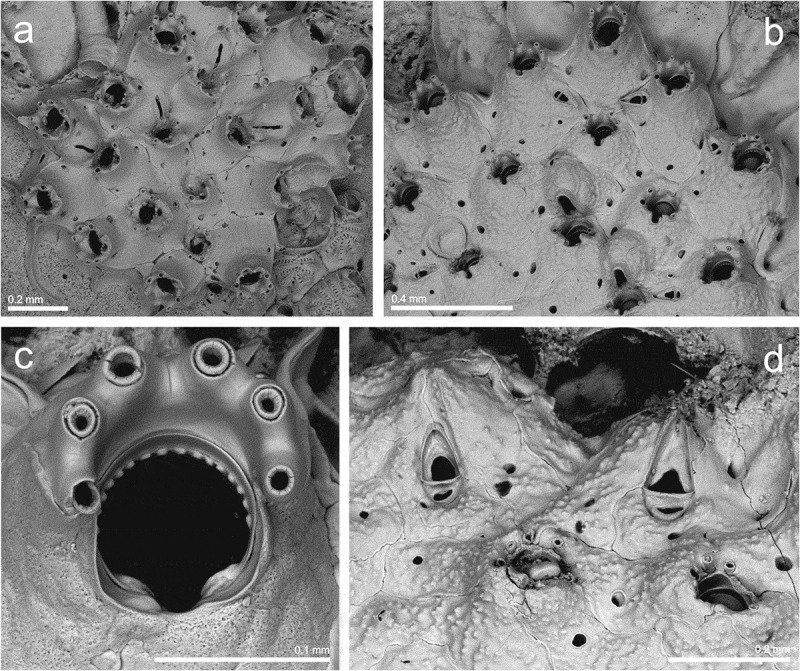
(a) Young colony of *Schizotheca divisa* (6a; MHNUSC-Bry 656); (b) ovicelled and non-ovicelled zooids and adventitious avicularia of *Schizotheca galaica* sp. nov. (st. 13; *Holotype*: MHNUSC 10091); (c) same, primary orifice; (d) same, vicarious avicularia.

Family **PHIDOLOPORIDAE** Gabb and Horn, 1862Genus ***Schizotheca*** Hincks, 1877***Schizotheca divisa*** (Norman, 1864)(Figure 6(a))

*Lepralia divisa* Norman, 1864: 86, pl. 10, fig. 6.

*Schizotheca divisa* (Norman): Hayward and Ryland 1999: 384, figs. 182, 183A, B.

#### Material examined

St. 6a: a small colony with embryos, on a broken shell (Ría de Ferrol: Redonda, 43.46389°N, 08.26333°W, 8 m depth; MHNUSC-Bry 656) (Figure 6(a)).

#### Description

Colony forming a small round incrustation of about 20 zooids. Autozooids oval, small, 0.264–0.294 (mean 0.279) mm long by 0.190–0.266 (mean 0.222) mm wide, separated by fine sutures. Frontal wall smooth, imperforate, except for two or three marginal pseudopores. Primary orifice broader than long, suborbicular; distal border finely denticulate. Peristome well developed, with an asymmetrical, deep U-shaped notch that may close distally, and a number of denticles in the inner side. Six oral spines, reduced to two or four in ovicellate zooids. Avicularia not present in the single colony observed. Ovicell flattened frontally, elongate, immersed by calcification of succeeding zooids, with a narrow median fissure, closed proximally. Ancestrula overgrown, not seen.

#### Remarks

According to Hayward and Ryland (1999) *S. divisa* is a rarely reported species, perhaps present only in the British Isles. However, the species was recently reported from the Netherlands by De Blauwe (2009). Therefore, the present record is the first one of *S. divisa* in Iberian waters, significantly expanding its geographical distribution to the south.

***Schizotheca galaica*** sp. nov.(Figure 6(b–d))

#### Type material

##### Holotype

MHNUSC 10091: St. 13: a small ovicelled colony on a dead fragment of *Porella compressa* together with more species; 43.36639°N, 09.32778°W, 223 m depth. (Figure 6(b–d)).

#### Etymology

Alluding to the presence of this species in Galicia (NW Iberian Peninsula).

#### Description

Colony unilaminar, forming a crust. Autozooids oval to rhomboidal, 0.318–0.374 (mean 0.345) mm long by 0.258–0.303 (mean 0.280) mm wide, in alternating series separated by fine grooves, sometimes indistinct; frontal surface slightly convex, granular, imperforate except for up to four large marginal pores. Primary orifice as long as wide, 0.085–0.090 (mean 0.088) mm long by 0.083–0.088 (mean 0.086) mm wide; anter semicircular, with 16 closely spaced, blunt denticulations, and poster with a shallow U-shaped sinus occupying one third of the proximal margin, with large condyles reaching the edges of the sinus. Peristome well developed, with a small, quadrate pseudosinus at the proximal border. Six oral spines on the edge of the colony, generally reduced to two to four in calcified zooids, and to two in ovicellate ones. An adventitious avicularium proximolaterally to the orifice, inconstant, 0.077–0.103 (mean 0.091) mm long by 0.041–0.050 (mean 0.046) mm wide; mandible triangular, orientated laterally; foramen extensive, occupying half to two-thirds of the rostrum; cross bar fine and without columella. Vicarious avicularia smaller than the autozooids, 0.184–0.214 (mean 0.199) mm long by 0.063–0.086 (mean 0.075) mm wide, situated particularly on the edges of the colony and orientated towards the periphery; structure similar to that of the adventitious avicularia. The vicarious avicularium is more than twice the size of the adventitious avicularium (≈ 2.2 times). Ovicell partially immersed by secondary calcification, 0.192–0.195 mm long by 0.155–0.156 mm wide, imperforate, with a parallel-sided proximal fissure. An ancestrula was not observed.

#### Remarks

*Schizotheca galaica* sp. nov. is closely similar to *Schizotheca carmenae* Reverter-Gil and Fernández-Pulpeiro, 2007b, a species described from the Azores and south Portugal. *Schizotheca galaica* sp. nov. differs from it most obviously by the shape of the primary orifice: in *S. carmenae* it is longer than wide, with anter semielliptical with about 12 roughly triangular denticulations, widely spaced, and with poster concave, without sinus, with two small round condyles (Reverter-Gil and Fernández-Pulpeiro 2007b, p. 1939, fig. 4C); in *S. galaica* sp. nov. the primary orifice is as wide as long, with anter semicircular with about 16 closely spaced, blunt denticulations, and poster with a shallow U-shaped sinus occupying one third of the proximal margin, with large condyles reaching the edges of the sinus (Figure 6(c)). Moreover, the autozooids and the avicularia, especially the adventitious one, are smaller in *S. galaica* sp. nov., and the shape of the proximal fissure of the ovicell is different in both species.

*Schizotheca galaica* sp. nov. shows some similarities with *Schizotheca fissa* (Busk, 1856), a species also present in Galicia, but differs most obviously by the presence of adventitious avicularia, absent in *S. fissa*.

**Figure 7.**
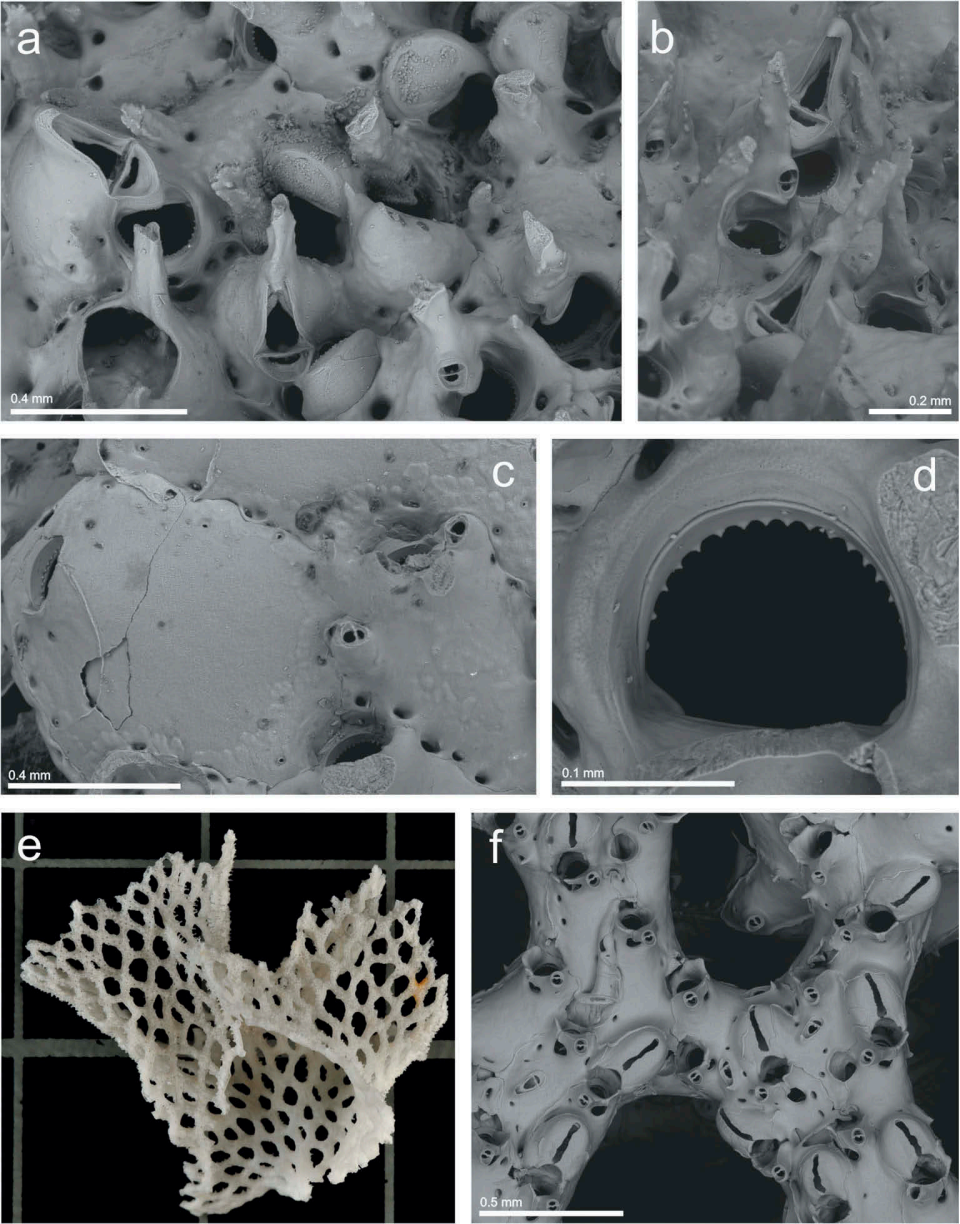
(a, b) Ovicelled zooids and avicularia of *Dentiporella saldanhai* (st. 15; MHNUSC-Bry 508); (c) same, edge of the colony and adventitious avicularia; (d) same, primary orifice; (e) a colony fragment of *Reteporella couchii* (st. 8; MHNUSC-Bry 501) (square = 1 mm^2^); (f) same, ovicelled zooids.

Genus ***Dentiporella*** Barroso, 1927***Dentiporella saldanhai*** Souto, Reverter-Gil and Fernández-Pulpeiro, 2010(Figure 7(a–d))

*Dentiporella saldanhai* Souto et al., 2010: 1429, fig. 9.

#### Material examined

St. 15: a large ovicelled colony on a dead coral (43.29139°N, 09.20667°W, 143 m depth; MHNUSC-Bry 508) (Figure 7(a–d)).

#### Description

Colony multilaminar, forming an extensive crust. Autozooids with thick frontal shield finely granular, bordered by a single series of marginal pores. Primary orifice D-shaped, as wide as long. Distal rim with 14–19 widely spaced denticles. Proximal rim straight to shallowly concave; small rounded condyles in the proximolateral corners. No oral spines. Peristome thick and deep, but not obscuring the primary one. A stout, columnar, median suboral umbo, spiky at the apex; the umbo bears an avicularium, facing towards the orifice and directed upwards. The avicularium may be either small, with oval rostrum, placed at the basis of the umbo; or large, with triangular rostrum distally hooked. A short uncinate process projects from the base of avicularia, facing a shallow lateral notch in the peristome. Small frontal oval avicularia are sporadically budded from the marginal pores. Ovicell globular, smooth, flat frontally, projecting vertically above peristome rim. Frontal ectooecium partly membranous.

#### Remarks

*Dentiporella saldanhai* was only recently described from material collected in the Algarve (S Portugal) at 19–20 m depth (Souto et al. 2010). As far as we are aware, the species was not reported again, and therefore the present record is not only the first one of *D. saldanhai* for the Galician coast but also the first one since the original description of the species.

Genus ***Reteporella*** Busk, 1884***Reteporella couchii*** (Hincks, 1878)(Figure 7(e,f))

*Retepora couchii* Hincks, 1878: 355, pl. 18, figs. 1–6.

*Reteporella couchii*: Hayward and Ryland 1999: 370, figs. 173, 174.

#### Material examined

St. 8: an ovicelled fragment, about 2 × 2 cm (43.54167°N, 08.43722°W, 94 m depth; MHNUSC-Bry 501) (Figure 7(e,f)).

St. 19: young colonies on a whale bone (42.80833°N, 09.39500°W, 128 m depth) (MHNUSC-Bry 403, 410).

#### Remarks

According to Hayward and Ryland (1999), *R. couchii* is a widespread species distributed in the Mediterranean, ranging northwards to west Norway and the Faroes, and southwards to the Gulf of Ghana. Off the Iberian Peninsula it has been reported in several localities in the Mediterranean, while along the Atlantic coast it was recorded only in the Algarve and in deep waters in the Cantabrian Sea. In Galicia, *R. couchii* had only been collected more than a century ago, off the north Galician coasts, by Jullien (1882, as *Retepora couchii*) at 1037 m depth and by Jullien and Calvet (1903, as *Sertella couchii*) at 300 m depth.

## Conclusions

A new species, *S. galaica* sp. nov., is described and new data on 18 additional species are here presented. Two species, *B. muriella* and *S. divisa*, are recorded for the first time from the Iberian Peninsula, and nine others (*A. longicollis, P. curvirostris, C. planum, G. corbula, H. sciaphilum, S. mamillata, F. asturiasensis, F. barrosoi* and *D. saldanhai*) are recorded for the first time in Galicia (NW Spain); for some of them (*A. galaica, F. asturiasensis, F. barrosoi* and *D. saldanhai*) these are also the first records since their original descriptions. Importantly, all these new data come from an area, Galicia, whose bryozoological fauna is considered to be the best known of the whole Iberian Peninsula, and one of the better known in Europe, with around 265 species known. This highlights the need to continue with systematic studies on biodiversity.

Half of the data presented here were obtained from the study of samples from the deep waters of the continental shelf, between *c*. 100 and 300 m, a range of depths that had barely been sampled before. Even though these depths of the continental shelf are mainly formed by sedimentary areas, which are not optimal for bryozoans, we have found 10 species here that were never found before in Galician water. This shows that small pieces of corals, shells, dead bryozoans, rocks, i.e. any substrate, can be colonised by bryozoans.

A special case is the Ría de Ferrol, from where we present three other new records (*T. inopinata, G. corbula* and *S. divisa*). This ría has been systematically surveyed since 30 years ago, and with more than 140 species, this is one of the areas with the greatest number of species per area of the entire Atlantic. Nonetheless, we continue to expect new discoveries in this area.

The general conclusion is, therefore, that it is worth continuing this work: new discoveries are still waiting.

**Table:** **Geolocation information**

Locality No	Locality name	Coordinates
1a	Ría de Ribadeo: R25	43.53694°N, 07.03611°W
1b	Ría de Ribadeo: R26	43.54000°N, 07.03750°W
1c	Ría de Ribadeo: R28	43.55000°N, 07.03583°W
1d	Ría de Ribadeo: R30	43.55222°N, 07.03694°W
2	Xove	43.71300°N, 07.46840°W
3	Ría de Ortigueira	43.70889°N, 07.83639°W
4	*Thalassa* Y428	44.19667°N, 08.67667°W
5a	Prior: El Porto	43.56000°N, 08.30300°W
5b	Prior: A Cova	43.56006°N, 08.31672°W
5c	Prior: A Cova	43.56006°N, 08.31672°W
6a	Ría de Ferrol: Redonda	43.46389°N, 08.26333°W
6b	Ría de Ferrol: Cetárea Vella	43.45592°N, 08.29939°W
6c	Ría de Ferrol: A Graña	43.47817°N, 08.25845°W
6d	Ría de Ferrol: Sta. Lucía	43.46139°N, 08.25000°W
7	Ría de Ares: Carnoedo	43.38083°N, 08.26361°W
8		43.54167°N, 08.43722°W
9	Malpica	43.32444°N, 08.81722°W
10		43.47806°N, 08.97083°W
11		43.39667°N, 09.18139°W
12		43.28194°N, 09.14333°W
13		43.36639°N, 09.32778°W
14		43.33917°N, 09.39556°W
15		43.29139°N, 09.20667°W
16		43.14500°N, 09.35361°W
17		43.25000°N, 09.45000°W
18		42.93333°N, 09.72833°W
19		42.80833°N, 09.39500°W
20a	Ría de Muros: Louro	42.75583°N, 09.10444°W
20b	Ría de Muros: P. San Francisco	42.75778°N, 09.07472°W
20c	Ría de Muros: Baroña	42.69194°N, 09.02833°W
21a	Ría de Arousa: N of O Grove	42.49167°N, 08.90833°W
21b	Ría de Arousa: O Grove	42.49610°N, 08.85920°W
21c	Ría de Arousa: Tragobe	42.51850°N, 08.82740°W
21d	Ría de Arousa: Cambados	42.52106°N, 08.84173°W
21e	Ría de Arousa: Illa de Arousa	42.55306°N, 08.93361°W
22a	Ría de Vigo: Cangas	42.25417°N, 08.83333°W
22b	Ría de Vigo: Canido	42.19361°N, 08.79917°W
22c	Ría de Vigo: V34	42.23889°N, 08.79639°W
23a	Cíes Islands: Illa de Monteagudo	42.24251°N, 08.90188°W
23b	Cíes Islands: Illa de Monteagudo	42.23871°N, 08.89947°W
23c	Cíes Islands: P. das Margaridas	42.23532°N, 08.89828°W
23d	Cíes Islands: P. Figueiras	42.23028°N, 08.89944°W
23e	Cíes Islands: Illa de Monteagudo	42.22883°N, 08.89511°W
23f	Cíes Islands: Illa San Martiño	42.20450°N, 08.90697°W

## References

[CIT0001] Álvarez JA. 1992 *Fenestrulina asturiasensis* sp. nov. (Bryozoa: Cheilostomida) from the northern coast of the Iberian Peninsula. J Mar Biol Assoc UK. 72:727–730. doi:10.1017/S002531540005949X

[CIT0002] Álvarez JA. 1993 *Fenestrulina barrosoi* sp. nov. (Bryozoa: Cheilostomida) with a review of the genus *Fenestrulina* on the Iberian Peninsula. J Mar Biol Assoc UK. 73:831–835. doi:10.1017/S0025315400034755

[CIT0003] Audouin JV 1826 Explication sommaire des planches de Polypes de l’Égypte et de la Syrie, publiées par Jules-César Savigny In: Panckoucke CLF, editor. (org.) Description de l’Égypte ou recueil des observations et des recherches qui ont été faites en Égyptes pendant l’Expédition de l’Armée française … Histoire naturelle. Tome 1 (4). Paris: Imprimerie Impériale; p. 225–244.

[CIT0004] Barroso MG 1923 Notas sobre los briozoos marinos españoles. XI. (Especies de Marín, Pontevedra). Bol R Soc Esp Hist Nat Biol. 23:119–126.

[CIT0005] Barroso MG 1927 Notas sobre Briozoos marinos españoles. Bol R Soc Esp Hist Nat Biol. 27:284.

[CIT0006] Berning B, Kukliński P 2008 North-east Atlantic and Mediterranean species of the genus *Buffonellaria* (Bryozoa, Cheilostomata): implications for biodiversity and biogeography. Zool J Linn Soc-Lond. 152:537–566. doi:10.1111/j.1096-3642.2007.00379.x

[CIT0007] Berning B, Tilbrook KJ, Rosso A 2008 Revision of the north-eastern Atlantic and Mediterranean species of the genera *Herentia* and *Therenia* (Bryozoa: Cheilostomata). J Nat Hist. 42:1509–1547. doi:10.1080/00222930802109140

[CIT0008] Bishop JDD, Househam BC 1987 *Puellina* (Bryozoa; Cheilostomatida; Cribrilinidae) from British and adjacent waters. Bull Brit Mus Nat Hist Zool. 53:1–63.

[CIT0009] Boury-Esnault N, Harmelin JG, Ledoyer M, Saldanha L, Zibrowius H 2001 Peuplement benthique des grottes sous-marines de Sagres (Portugal, Atlantique nordoriental). Bol Mus Municip Funchal. Suplemento. 6:13–35.

[CIT0010] Busk G 1852 Catalogue of marine Polyzoa in the collection of the British Museum. I. Cheilostomata. London: Trustees of the British Museum (Natural History). doi:10.5962/bhl.title.20859

[CIT0011] Busk G 1856 Zoophytology. Q J Microsc Sci. 4:308–312.

[CIT0012] Busk G 1859 A monograph of the fossil Polyzoa of the Crag. London: The Palaeontographical Society.

[CIT0013] Busk G 1884 Report on the Polyzoa collected by H.M.S. Challenger during the years 1873–1876. Part 1. The Cheilostomata. Rep Sci Result Voyage HMS “Challenger”, Zool. 10:1–216.

[CIT0014] Canu F, Bassler RS 1917 A synopsis of American early Tertiary Cheilostome Bryozoa. US Nat Mus Bull. 96:1–87.

[CIT0015] Canu F, Bassler RS 1927 Classification of the cheilostomatous Bryozoa. Proc US Nat Mus. 69:1–42. doi:10.5479/si.00963801.69-2640.1.

[CIT0016] Carrada GC 1973 Briozoi litorali della Ria di Vigo (Spagna Nord-Occidentale). Invest Pesq. 37:9–15.

[CIT0017] César-Aldariz J, Fernández-Pulpeiro E, Reverter-Gil O 1999 A new species of the genus *Celleporella* (Bryozoa: Cheilostomatida) from the European Atlantic coast. J Mar Biol Assoc UK. 79:51–55. doi:10.1017/S0025315498000058

[CIT0018] d’Hondt JL 1970 Campagne d’essais du Jean Charcot (3-8 decembre 1968). 5. Bryozoaires. Bull Mus Nat Hist Nat (2^e^ Ser). 42:232–256.

[CIT0019] d’Hondt JL 1974 Bryozoaires récoltés par la «Thalassa» dans le Golfe de Gascogne (Campagnes de 1968 à 1972). Cah Biol Mar. 15:27–50.

[CIT0020] d’Hondt JL 1988 Bryozoaires marins du Guipúzcoa. Cah Biol Mar. 29:513–529.

[CIT0021] d’Hondt JL, Occhipinti AA 1985 *Tricellaria inopinata* n.sp., un nouveau Bryozoaire Cheilostome de la faune Méditerrannée. Pubb Staz Zool Napoli (Mar Ecol). 6:35–46.

[CIT0022] d’Orbigny A 1851–54. Paléontologie française. Description des Mollusques et Rayonnés fossiles. Terrains crétacés, V. Bryozoaires. Paris: Victor Masson.

[CIT0023] David L, Pouyet S 1978 Le genre *Herentia* Gray, 1848 (Bryozoa, Cheilostomata). Systématique et phylogenèse, biostratigraphie et biogéographie. Doc Lab Géol Fac Sci Lyon HS. 4:167–193.

[CIT0024] De Blauwe H 2009 Mosdiertjes van de Zuidelijke bocht van de Noordzee: determinatiewerk voor België en Nederland. Oostende (Belgium): Vlaams Instituut voor de Zee (VLIZ) http://www.vliz.be/NL/home/&p=show&id=486.

[CIT0025] Ellis J, Solander DC 1786 The natural history of many curious and uncommon zoophytes, collected from various parts of the globe. London: White and Elmsly.

[CIT0026] Fernández-Pulpeiro E, César-Aldariz J, Reverter-Gil O 2002 Sobre la presencia de *Tricellaria inopinata* d’Hondt y Occhipinti Ambrogi, 1985 (Bryozoa, Cheilostomatida) en el litoral gallego (N.O. España). Nov Acta Cient Compos Biol. 11:207–213. http://hdl.handle.net/10347/6631.

[CIT0027] Fleming J 1828 A history of British animals, exhibiting their descriptive characters and systematical arrangement of the genera and species of quadrupeds, birds, reptiles, fishes, Mollusca, and Radiata of the United Kingdom. Edinburgh: Bell and Bradfute.

[CIT0028] Gabb WM, Horn GH 1862 The fossil Polyzoa of the secondary and tertiary formations of North America. J Acad Nat Sci Philadelphia. 5:111–179.

[CIT0029] Gautier Y-V 1962 Recherches écologiques sur les Bryozoaires Chilostomes en Méditerranée occidentale. Rec Trav St Mar Endoume. 38:1–434.

[CIT0030] Gray JE 1848 List of the specimens of British animals in the collection of the British Museum. Part 1. Centroniae or radiated animals. London: Trustees of the British Museum (Natural History).

[CIT0031] Harmelin JG, Arístegui J 1988 New Cribrilinidae (Bryozoa, Cheilostomata) from the upper bathyal of the Atlanto-Mediterranean region. J Nat Hist. 22:507–535. doi:10.1080/00222938800770351

[CIT0032] Harmelin JG, D’ Hondt JL 1992 Bryozoaires des parages de Gibraltar (campagne océanographique BALGIM, 1984) 1 – chéilostomes. Bull Mus Nat Hist Nat Zool Biol Ecol Anim. 14:23–67.

[CIT0033] Harmer SF 1926 The Polyzoa of the Siboga Expedition. Part 2. Cheilostomata Anasca. Siboga Expeditie. 28b:181–501.

[CIT0034] Hayward PJ, McKinney FK 2002 Northern Adriatic Bryozoa from the vicinity of Rovinj, Croatia. Bull Am Mus Nat Hist. 270:1–139. http://digitallibrary.amnh.org/dspace/handle/2246/486.

[CIT0035] Hayward PJ, Ryland JS 1998 Cheilostomatous Bryozoa. Part 1. Aeteoidea-Cribrilinoidea. Synopses of the British Fauna (New Series). Vol. 10, 2nd ed. London: Linnean Society of London and The Estuarine and Brackish-water Science Association p. 1–366

[CIT0036] Hayward PJ, Ryland JS 1999 Cheilostomatous Bryozoa. Part 2. Hippothoidea-Celleporoidea. Synopses of the British Fauna (New Series). Vol. 14, 2nd ed. London: Linnean Society of London and The Estuarine and Brackish-water Science Association p. 1–416

[CIT0037] Hincks T 1862 Catalogue of the Zoophytes of South Devon and Cornwall. Ann Mag Nat Hist. 9:22–30, 200–207, 303–310, 467–475. doi:10.1080/00222936208681181.

[CIT0038] Hincks T 1877 On British Polyzoa Part II. Classification. Ann Mag Nat Hist. 20:520–532. doi:10.1080/00222937708682275.

[CIT0039] Hincks T 1878 Notes on the genus *Retepora*, with descriptions of new species. Ann Mag Nat Hist. 1:353–365. doi:10.1080/00222937808682345.

[CIT0040] Hincks T 1879 On the classification of the British Polyzoa. Ann Mag Nat Hist. 3:153–164. doi:10.1080/00222937908682494.

[CIT0041] Hincks T 1880 A history of the British marine Polyzoa. Vol. 2, London: van Voorst.

[CIT0042] Hughes RN, Gómez A, Wright PJ, Moyano HI, Cancino JM, Carvalho GR, Lunt DH 2008 Molecular phylogeny supports division of the ‘cosmopolitan’ taxon *Celleporella* (Bryozoa; Cheilostomata) into four major clades. Mol Phylogenet Evol. 46:369–374. doi:10.1016/j.ympev.2007.12.020. 17931893

[CIT0043] Johnston G 1838 A History of the British Zoophytes. Edinburgh: W.H. Lizars.

[CIT0044] Johnston G 1847 A History of the British Zoophytes. London: Van Voorst.

[CIT0045] Jullien J 1882 Dragages du « Travailleur ». Bryozoaires. Espèces draguées dans l’Océan Atlantique en 1881. Espèces nouvelles ou incomplètement décrites. Extrait Bull Soc Zool Fr. 7:1–33+5 pls. doi:10.5962/bhl.title.4721

[CIT0046] Jullien J 1883 Dragages du « Travailleur », Bryozoaires. Espèces draguées dans l’Océan Atlantique en 1881. (Séance du 26 décembre 1882). Espèces nouvelles ou incomplètement décrites. Bull Soc Zool Fr. 7:497–534.

[CIT0047] Jullien J 1888 Bryozoaires. Mission Scientifique Du Cap Horn. 1882–1883:61–92.

[CIT0048] Jullien J, Calvet L 1903 Bryozoaires provenant des Campagnes de « l’Hirondelle » (1886-1888). Résultats des Campagnes Scientifiques accomplies sur son yacht, par Albert I. 23:1–188.

[CIT0049] Lamouroux JVF 1812 Extrait d’un mémoire sur la classification des Polypiers coralligènes non entièrement pierreux. Nouv Bull Sci Soc Philos. 3:181–188.

[CIT0050] Lamouroux JVF 1816 Histoire des Polypiers Coralligènes Flexibles, vulgairement nommés Zoophytes. Caen: F. Poisson.

[CIT0051] Levinsen GMR 1909 Morphological and systematic studies on the Cheilostomatous Bryozoa. Copenhagen: Nationale Forfatterers Forlag.

[CIT0052] Linnaeus C 1758 Systema Naturae per Regna Triae Naturae, secundum classes, ordines, genera, species, cum characteribus, differentiis, synonymis, locis. 10 ed. Holmiae: Laurentii Salvii.

[CIT0053] MacGillivray PH 1895 A monograph of the Tertiary Polyzoa of Victoria. T Roy Soc Victoria. 4:1–166.

[CIT0054] Michelin H 1848 Iconographie zoophytologique, description par localités et terrains des Polypiers fossiles de France et pays environnants. Paris: Bertrand édit.

[CIT0055] Moyano GHI 1987 Briozoos Marinos Chilenos VI. Cheilostomata Hippothoidae: south Eastern Pacific species. Bol Soc Biol Concepción. 57:89–135.

[CIT0056] Norman AM 1864 On undescribed British Hydrozoa, Actinozoa and Polyzoa. Ann Mag Nat Hist. 13(3):82–90. doi:10.1080/00222936408681578

[CIT0057] Norman AM 1903 Notes on the natural history of East Finmark. Polyzoa. Ann Mag Nat Hist. 11(7):567–598. doi:10.1080/00222930308678818

[CIT0058] Osburn RC 1940 Bryozoa of Porto Rico with a resume of West Indian Bryozoan fauna. Ann NY Acad Sci. 16:321–486.

[CIT0059] Prenant M, Bobin G 1966 Bryozoaires, 2^ème^ partie. Chilostomes Anasca. Faune Fr. 68:1–647.

[CIT0060] Reverter-Gil O 1995 Briozoos de la Ría de Ferrol [PhD thesis]. Santiago de Compostela: University of Santiago de Compostela.

[CIT0061] Reverter O, Fernández E 1996 Cribrilinidae (Bryozoa: Cheilostomatida) from the Ria de Ferrol (NW Spain). J Nat Hist. 30:1247–1260. doi:10.1080/00222939600770681.

[CIT0062] Reverter-Gil O, Fernández-Pulpeiro E 2001 Inventario y cartografía de los Briozoos marinos de Galicia (N.O. de España). Nova Acta Cient Compos, Monogr. 1:1–243.

[CIT0063] Reverter-Gil O, Fernández-Pulpeiro E 2007a A new name for *Puellina parva* Reverter and Fernández, 1996 (Bryozoa, Cheilostomatida). J Nat Hist. 41:729–730. doi:10.1080/00222930701261810

[CIT0064] Reverter-Gil O, Fernández-Pulpeiro E 2007b Species of genus *Schizotheca* Hincks (Bryozoa, Cheilostomata) described in the Atlantic-Mediterranean region, with notes on some species of *Parasmittina* Osburn. J Nat Hist. 41:1929–1953. doi:10.1080/00222930701515520

[CIT0065] Reverter-Gil O, Souto J, Fernández-Pulpeiro E 2014 Annotated checklist of recent marine Bryozoa from continental Portugal. Nov Acta Cient Compos Biol. 21:1–55. http://www.usc.es/revistas/index.php/nacc/issue/view/208.

[CIT0066] Reverter-Gil O, Souto J, Novosel M, Tilbrook KJ 2015 Adriatic species of *Schizomavella* (Bryozoa: Cheilostomata). J Nat Hist. doi:10.1080/00222933.2015.1062153

[CIT0067] Rosso A, Beuck L, Vertino A, Sanfilippo R, Freiwald A 2018 Cribrilinids (Bryozoa, Cheilostomata) associated with deep-water coral habitats at the Great Bahama Bank slope (NW Atlantic), with description of new taxa. Zootaxa. 4524:401–439. doi:10.11646/zootaxa.4524.4. 30486103

[CIT0068] Silén L, Harmelin JG 1976 *Haplopoma sciaphilum* sp. n., a cave-living bryozoan from the Skagerrak and the Mediterranean. Zool Scr. 5:61–66. doi:10.1111/j.1463-6409.1976.tb00682.x.

[CIT0069] Smitt FA 1868 Kritisk Förteckning öfver Skandinaviens Hafs-Bryozoer: pt III. Öfvers Kongl Vetensk-Akad Förh. 24:279–429.

[CIT0070] Smitt FA 1873 Floridan Bryozoa, collected by Count L.F. de Pourtales. Part II. K Sven Vetensk Akad Handl. 11:1–83.

[CIT0071] Soto García E, Fernández Pulpeiro E, Ramil Blanco F 2002 Briozoos infralitorales de la Ría de Vigo (España). Bol R Soc Esp Hist Nat Biol. 97:85–96.

[CIT0072] Souto J, Reverter-Gil O, De Blauwe H, Fernández-Pulpeiro E 2014 New records of Bryozoans from Portugal. Cah Biol Mar. 55:129–150.

[CIT0073] Souto J, Reverter-Gil O, Fernández-Pulpeiro E 2010 Gymnolaemate Bryozoans from the Algarve (S. Portugal). New species and biogeographical considerations. J Mar Biol Assoc UK. 90(7):1417–1439. doi:10.1017/S0025315409991640

[CIT0074] Tilbrook KJ 2006 Cheilostomatous Bryozoa from the Solomon Islands. Santa Barbara Mus Nat Hist. Monographs 4(Studies in Biodiversity Number 3):1–386.

[CIT0075] Vieira LM, Spencer Jones ME 2012 The identity of *Sertularia reptans* Linnaeus, 1758 (Bryozoa, Candidae). Zootaxa. 3563:26–42. http://www.mapress.com/zootaxa/list/2012/3563.html.

[CIT0076] Vieira LM, Spencer Jones ME, Winston JE 2013 *Cradoscrupocellaria*, a new bryozoan genus for *Scrupocellaria bertholletii* (Audouin) and related species (Cheilostomata, Candidae): taxonomy, biodiversity and distribution. Zootaxa. 3707:1–63. doi:10.11646/zootaxa.3707.1.1 26146678

[CIT0077] Zabala M 1986 Fauna dels Briozous dels Països Catalans. Institut d’Estudis Catalans. Arxius de la Secció de Ciències. 84:1–833.

[CIT0078] Zabala M 1993 Els Briozous In: Alcover JA, Ballesteros E, Fornos JJ, editors. Història Natural de l’Arxipèlag de Cabrera. Palma de Mallorca: Monografies de la Societat d’Història Natural de les Balears; p. 561–577.

